# Implementation strategies to improve cervical cancer prevention in sub-Saharan Africa: a systematic review

**DOI:** 10.1186/s13012-018-0718-9

**Published:** 2018-02-09

**Authors:** Lauren G. Johnson, Allison Armstrong, Caroline M. Joyce, Anne M. Teitelman, Alison M. Buttenheim

**Affiliations:** School of Nursing, University of Pennsylvania, Philadelphia, PA USA

**Keywords:** Cervical cancer, Prevention, Program implementation, Implementation strategies, Sub-Saharan Africa

## Abstract

**Background:**

Developed countries, such as the USA, have achieved significant decreases in cervical cancer burden since the introduction of Pap smear-based programs in the 1960s. Due to implementation barriers and limited resources, many countries in sub-Saharan Africa (SSA) have been unable to attain such reductions. The purpose of this review is to evaluate implementation strategies used to improve the uptake and sustainability of cervical cancer prevention programs in SSA.

**Methods:**

A reviewer (LJ) independently searched PubMed, Ovid/MEDLINE, Scopus, and Web of Science databases for relevant articles with the following search limits: English language, peer reviewed, and published between 1996 and 2017. The 4575 search results were screened for eligibility (CJ, LJ) to identify original research that empirically evaluated or tested implementation strategies to improve cervical cancer prevention in SSA. Fifty-three articles met criteria for inclusion in the final review. AA, CJ, and LJ abstracted the included articles for implementation-related content and evaluated them for risk of bias according to study design with the National Heart, Lung, and Blood Institute’s (NHLBI) Quality Assessment Tools. Results were reported according to PRISMA guidelines.

**Results:**

The 53 included studies are well represented among all sub-Saharan regions: South (*n* = 16, 30.2%), West (*n* = 16, 30.2%), East (*n* = 14, 26.4%), and Middle (*n* = 7, 13.2%). There are 34 cross-sectional studies (64.2%), 10 pre-posttests (18.9%), 8 randomized control trials (15.1%), and one nonrandomized control trial (1.9%). Most studies are “fair” quality (*n* = 22, 41.5%). Visual inspection with acetic acid (VIA) (*n* = 19, 35.8%) was used as the main prevention method more frequently than HPV DNA/mRNA testing (*n* = 15, 28.3%), Pap smear (*n* = 13, 24.5%), and HPV vaccine (*n* = 9, 17.0%). Effectiveness of strategies to improve program implementation was measured using implementation outcomes of penetration (*n* = 33, 62.3%), acceptability (*n* = 15, 28.3%), fidelity (*n* = 14, 26.4%), feasibility (*n* = 8, 15.1%), adoption (*n* = 6, 11.3%), sustainability (*n* = 2, 3.8%), and cost (*n* = 1, 1.9%). Education strategies (*n* = 38, 71.7%) were used most often but have shown limited effectiveness.

**Conclusion:**

This systematic review highlights the need to diversify strategies that are used to improve implementation for cervical cancer prevention programs. While education is important, implementation science literature reveals that education is not as effective in generating change. There is a need for additional organizational support to further incentivize and sustain improvements in implementation.

**Electronic supplementary material:**

The online version of this article (10.1186/s13012-018-0718-9) contains supplementary material, which is available to authorized users.

## Background

Cervical cancer is a high-burden global health issue, with an estimated 528,000 new cases and 266,000 deaths in 2012 for women across the world [1]. Most of the global burden (85%) lies in less developed countries, with regions in sub-Saharan Africa (SSA) having the largest age-standardized incidence and mortality rates [1]. Developed countries, such as the USA, have achieved significant decreases in cervical cancer burden since the introduction of organized Pap smear programs in the 1960s [2, 3]. However, many countries in SSA have been unable to attain such reductions due to implementation barriers and resource limitations [4–8]. In fact, cervical cancer rates are expected to continue rising despite efforts to implement national screening and treatment programs [9]. Cervical cancer remains the most commonly diagnosed cancer and leading cause of cancer-related death in African women south of the Sahara [1].

Untangling the causes for high cervical cancer burden in SSA is difficult due to a complex interplay of many biological, organizational, economic, and sociocultural factors. For example, HIV has been correlated with an increased risk for developing cervical cancer [10]. HIV infection causes the body to become immunocompromised and more susceptible to contracting HPV, which is a significant precursor to cervical cancer [10]. SSA incidentally carries a high HIV/AIDS burden, accounting for 71% of the global population living with HIV [11]. Furthermore, young women bear a disproportionate HIV burden compared to their male peers [11]. Other contributory factors include the aging and growth of the population, limited access to medical facilities, poor nutrition, severity of disease at presentation, and insufficient facilities for treatment [12–15]. While these factors contribute to the rise in cervical cancer for this region, this paper focuses on the need for improved implementation of existing prevention programs and the promise that increased access to preventive services has on decreasing burden.

Prevention is key. With adequate resources, precancerous cervical lesions are easily prevented and treatable [16, 17]. The incubation period between HPV infections developing into cervical cancer is 10 to 20 years, which allows ample opportunities to screen, track, and treat across the disease progression [18]. In addition, numerous technologies have been developed to detect and treat precancerous lesions including Pap smear, colposcopy, visual inspection with acetic acid or Lugol’s iodine (VIA/VILI), HPV DNA testing, cone biopsy, cryotherapy, and loop electrosurgical incision procedure (LEEP) [2, 19]. Although these tools have been proven safe and effective [20], there are still significant challenges in implementing them into comprehensive national screening and treatment programs.

For decades, developed countries have used cytology-based programs with Pap smear as the standard screening protocol [2, 3, 8]. However, these programs require lab infrastructure that is not readily available in many SSA countries and is often prohibitively expensive to sustain on a large scale [21]. Alternative screening methods have been developed with the hope of being more sustainable in resource-limited settings [8]. Visual inspection with acetic acid and Lugol’s iodine (VIA/VILI) are visual tests that are used to identify precancerous lesions with the naked eye. VIA and VILI are advantageous because they can be performed by non-physician providers (addressing provider shortages) and provide immediate results (reducing loss to follow-up) [22–24]. VIA and VILI have similar sensitivity when compared to Pap smear and can provide screening at a much lower cost and with fewer staff needed [20, 24, 25]. However, these visual tests are less specific and can lead to overtreatment [20, 24]. HPV DNA testing is another alternative screening method that is used to identify high risk, carcinogenic HPV (typically types 16 and 18). The test can be performed at home with self-sampling kits and has been acceptable for many surveyed women [26–31]. It can also be used as a preliminary triage to save time and resources on women that screen HPV negative and do not require follow-up testing [32, 33]. HPV DNA testing does not require the same level of lab infrastructure as Pap smear, but it involves lab processing nonetheless and wait times to receive results [8].

Despite development of alternatives to Pap smear, a significant research-to-practice gap still exists. Lack of trained providers, overburdened health facilities, insufficient supplies, inadequate lab infrastructure, loss to treatment follow-up, high costs, and cultural beliefs are some of the implementation barriers experienced in SSA [4–8]. In addition to seeking alternative screening methods, SSA countries can further improve their prevention efforts by developing and employing implementation strategies to overcome these barriers. An implementation strategy is defined as “a systematic intervention process to adopt and integrate evidence-based health innovations into usual care” [34]. The purpose of this systematic review is to uncover the breadth and diversity of implementation strategies used to improve the uptake and sustainability of cervical cancer prevention programs in SSA. Through highlighting different strategies, we aim to assist researchers, practitioners, managers, and policy makers in scaling up and evaluating new and existing programs.

## Methods

### Search strategy

**Fig. 1:**
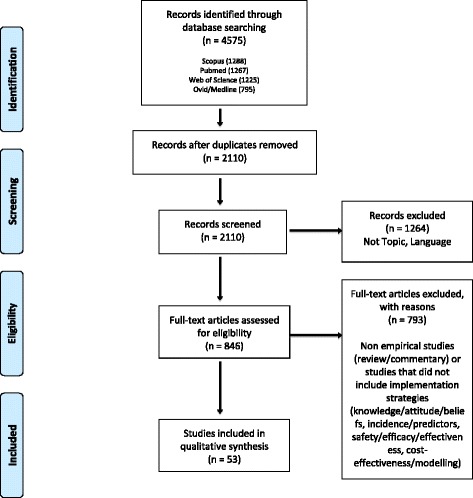
Search strategy. The following search strategy is reported according to PRISMA guidelines

Figure 1 outlines the search strategy, which has been reported according to Preferred Reporting Items for Systematic Reviews and Meta-Analyses (PRISMA) guidelines [35, 36]. A reviewer (LJ) independently searched PubMed, Ovid/MEDLINE, Scopus, and Web of Science databases with the following approximate search terms: (cervical cancer OR HPV) AND (prevention OR screening OR program OR implementation OR scale-up OR Pap smear OR VIA OR VILI OR see-and-treat OR HPV vaccine OR HPV DNA test OR self-sampling OR colposcopy OR cryotherapy OR LEEP) AND (sub-Saharan Africa OR country-specific terms for each SSA country). Search strategies with specific terminology for each database are included as Additional file 1.

### Eligibility criteria

Inclusion and exclusion criteria were developed to identify original research that empirically evaluated or tested implementation strategies to improve cervical cancer prevention in SSA. Articles were eligible for inclusion if written in English, peer reviewed, and published between 1996 and 2017. Non-empirical studies (reviews, commentaries, editorials, etc.) and studies that did not explicitly assess implementation strategies (knowledge, attitudes, and beliefs; incidence and prevalence; safety and efficacy; cost effectiveness and modeling) were excluded from the review.

### Study selection

The initial database search yielded 4575 results. Two reviewers (CJ, LJ) conducted the study selection process. Titles and abstracts of the identified articles were screened to exclude duplicates (*n* = 2465) and studies not relevant to the topic (*n* = 1264). The remaining articles (*n* = 846) were reviewed in full text. Fifty-three studies met the eligibility criteria and an additional 793 articles were excluded.

### Data extraction

The 53 articles that fit the inclusion criteria were extracted for the following implementation-related content: title, author, publication year, purpose, country, study design, prevention tools, implementation strategies, implementation outcomes, and results. The primary reviewer (LJ) and two additional reviewers (AA, CJ) completed data extraction for a sample of initial articles (*n* = 11, 20%) to ensure accuracy. Inconsistencies were resolved through consensus before the primary reviewer proceeded with the remaining articles. Results were summarized in frequency tables.

Two seminal articles from implementation science, Proctor et al. [37] and Powell et al. [34], were used to define and categorize implementation outcomes and strategies, respectively. Based on the Conceptual Model of Implementation Research [38], Proctor et al. developed a taxonomy of implementation outcomes that are conceptually distinct from service system outcomes and clinical treatment outcomes. Implementation outcomes were defined as “the effects of deliberate and purposive actions to implement new treatments, practices, and services.” Using an iterative process of reading and discussing relevant literature in behavioral and health science, the working group of implementation researchers defined eight implementation outcomes: acceptability, adoption, appropriateness, costs, feasibility, fidelity, penetration, and sustainability. Powell et al. used the Consolidated Framework for Implementation Research [39] to compile a list of implementation strategies, or “systematic intervention processes to adopt and integrate evidence-based health innovations into usual care.” A working group of researchers and clinicians from health and mental services used narrative review to develop six categories: educate, restructure, quality, finance, plan, and attend to policy context. A complete list of categories and their definitions for implementation outcomes and strategies can be found in Table 1.

**Table 1:** Implementation outcomes and strategies

Implementation outcome	Definition^a^
Acceptability	Perception among implementation stakeholders that a given treatment, service, practice, or innovation is agreeable, palatable, or satisfactory
Adoption	Intention, initial decision, or action to try or employ an innovation or evidence-based practice
Appropriateness	Perceived fit, relevance, or compatibility of the innovation or evidence based practice setting, provider, or consumer; and/or perceived fit of innovation to address a particular issue
Penetration	Integration of a practice within a service setting and its subsystems; number of eligible persons who use a service, divided by the total number of persons eligible for the service; number of providers who deliver a given service or treatment, divided by the total number of providers trained in or expected to deliver the service
Feasibility	Extent to which a new treatment, or an innovation, can be successfully used or carried out within a given agency or setting
Fidelity	Degree to which an intervention was implemented as it was prescribed in the original protocol or as it was intended by the program developers
Sustainability	Extent to which a newly implemented treatment is maintained or institutionalized within a service setting’s ongoing, stable operations
Implementation cost	Cost impact of an implementation effort
Implementation strategy	Definition^a^
Plan • Gather information • Build buy-in • Initiate leadership • Develop relationships	Help stakeholders gather data, select strategies, build buy-in, initiate leadership, and develop the relationships necessary for successful implementation
Educate • Develop materials • Inform and influence stakeholders	Inform a range of stakeholders about the innovation and/or implementation effort
Finance • Modify incentives • Facilitate financial support	Incentivize the use of clinical innovations and provide resources for training and ongoing support.
Restructure	Facilitate implementation by altering staffing, professional roles, physical structures, equipment, and data systems
Quality management	Put data systems and support networks in place to continually evaluate and enhance quality of care, and ensure that clinical innovations are delivered with fidelity
Attend to policy context	Encourage the promotion of clinical innovations through accrediting bodies, licensing boards, and legal system

^a^Definitions for implementation outcomes and strategies have been cited in Proctor et al. [37] and Powell et al. [34], respectively

### Quality screening

Quality assessment tools from the National Heart, Lung, and Blood Institute (NHLBI) were used to assess each study for internal validity [40]. There are separate NHLBI Quality Assessment Tools for each study type (controlled trials, pre-posttest, and cross-sectional). Each tool includes specific questions to assess bias, confounding, power, and strength of association between intervention and outcomes. The answer to each question can be “yes,” “no,” “cannot determine,” “not reported,” or “not applicable.” Instead of using a numeric scoring system, the rater is asked to consider potential risk for bias in the study design whenever a “no” is selected. Overall quality ratings are scored as “good” (low risk of bias, valid results), “fair” (some risk of bias, does not invalidate results), or “poor” (significant risk for bias, may invalidate results). One reviewer (LJ) independently screened all studies, and two additional reviewers (AA, CJ) screened a 20% sample (*n* = 11) to double check for accuracy.

## Results

Of the initial 4575 articles (2110 after duplicates removed), 53 met inclusion criteria and were included in the following synthesis of results. Study characteristics are summarized in Table 2. The table of evidence is included as Table 3. Most studies were published within the last 7 years. Studies were well represented in all regions of sub-Saharan Africa with 16 of the total studies (30.2%) conducted in Southern Africa, 16 (30.2%) in Western, 14 (26.4%) in Eastern, and 7 (13.2%) in Middle.

**Table 2:** Study characteristics

	Number of studies (*n*)	Percentage of total studies (%)
Sub-Saharan region		
South	16	30.2
West	16	30.2
East	14	26.4
Middle	7	13.2
Country		
South Africa	14	26.4
Nigeria	10	18.9
Cameroon	7	13.2
Kenya	5	9.4
Uganda	4	7.5
Ghana	3	5.7
Botswana	2	3.8
Tanzania	1	1.9
Cote d’Ivoire	1	1.9
Zambia	1	1.9
Gambia	1	1.9
Mozambique	1	1.9
Malawi	1	1.9
Madagascar	1	1.9
Mali	1	1.9
Publication date		
1996–2000	1	1.9
2001–2005	2	3.8
2006–2010	8	15.1
2011–2017	42	79.2
Study design		
Cross-sectional	34	64.2
Pre-post test	10	18.9
Randomized control trial	8	15.1
Nonrandomized control trial	1	1.9
Prevention tool		
VIA	19	35.8
HPV DNA or RNA test	15	28.3
Pap smear	13	24.5
HPV vaccine	9	17.0
Digital imaging	9	17.0
VILI	9	17.0
Colposcopy	7	13.2
Cryotherapy	5	9.4
LEEP	5	9.4
Biopsy	5	9.4
Unspecified screening	5	9.4
Implementation strategy		
Educate	38	71.7
Restructure	26	49.1
Quality	13	24.5
Finance	5	9.4
Plan	1	1.9
Attend to policy context	0	0.0
Implementation outcome		
Penetration	33	62.3
Acceptability	15	28.3
Fidelity	14	26.4
Feasibility	8	15.1
Adoption	6	11.3
Sustainability	2	3.8
Cost	1	1.9
Appropriateness	0	0.0
Quality assessment		
Poor	20	37.7
Fair	22	41.5
Good	11	20.8

**Table 3:** Table of evidence

First author, year	Purpose	Country	Program	Strategy	Outcome	Results	Quality
Randomized control trials							
Adonis 2017 [79]	To evaluate what type of framed email messaging has the best impact on Pap smear uptake among health-insured females	South Africa	Pap smear	Educate: educational email	Penetration: Pap smear screening coverage	Screening rate in the control group was 9.58%, 5.71% in the gain-framed group, and 8.53% in the loss-framed group. Statistically, there was no difference between groups.	Fair
Modibbo 2017 [82]	To investigate whether self-collection of cervicovaginal samples for HPV DNA tests would be associated with increased uptake and quality of screening compared with clinic-based collection of samples	Nigeria	HPV DNA test	Restructure: remote self-collection vs. clinic-based physician collection	Fidelity: sensitivity and specificity between clinician- and self- collected samples	Most participants in the self-collection arm (93%) submitted their samples while only 56% of those invited to the hospital for sample collection attended and were screened during the study period (*p* < 0.001)	Fair
Okeke 2013 [80]	Determine the effect of cost on screening uptake by providing randomly priced subsidies to eligible women	Nigeria	VIA	Finance: lottery for varied prices of screening and treatment subsidies (0, 50, and 100 Naira)	Penetration: VIA screening coverage	Price of screening had a significant effect on the demand for screening: reducing the price by 10 cents increased uptake by 1%.	Fair
Risi 2004 [77]	Evaluate the effectiveness of two media interventions—a photo-comic and a radio-drama—in increasing cervical screening uptake	South Africa	Pap smear	Educate: educational photo-comic and radio-drama	Penetration: Pap smear screening coverage	7% (18 of 269) of women who received the intervention photo-comic reported cervical screening during the 6-month follow-up, compared with 6% (25 of 389) of controls (*p* = 0.89). Women who recalled hearing the radio-drama were more likely to report attending screening (9 of 53, 17%) than those who did not (19 of 429, 4%; *p* < 0.001).	Good
Rosser 2015 [78]	Evaluate a health talk’s impact on cervical cancer knowledge, attitudes, and screening rates in a rural setting	Kenya	Unspecified screening	Educate: 30-min didactic lecture	Acceptability: reasons for refusal Adoption: willingness to screen Penetration: screening coverage	Mean knowledge scores increased by 26.4% in the intervention arm compared to only 17.6% in the control arm (*p* < 0.01). Screening uptake was moderate in both the intervention and control arms, with no difference between the groups (58.9 vs. 60.9%, *p* = 0.60).	Fair
Sossaeuer 2014 [26]	Evaluate whether an educational intervention would improve women’s knowledge and confidence in the Self-HPV method	Cameroon	HPV DNA test	Educate: individual counseling (all), educational video (intervention group	Acceptability: confidence, embarrassment, pain, anxiety, discomfort, degree of relaxation and confidence	Participants who received the educational intervention had significantly higher knowledge about HPV and cervical cancer than the control group but no significant difference on self-HPV acceptability and confidence in the method.	Fair
Van Wijgert 2006 [29]	Assess the validity, feasibility, and acceptability of two methods of self-sampling (tampon or vaginal swab) compared to clinician sampling during a speculum examination	South Africa	HPV DNA test	Restructure: self-administration with tampon or vaginal swab vs. clinician collected swabs	Acceptability: perceived pain, satisfaction Feasibility: proportion of invalid labs Fidelity: sensitivity and specificity between clinician-and self-collected samples	Sensitivity for high-risk HPV was good for vaginal swabs (79.8%) and moderate for tampons (59.5%). Self- and clinician- sampling were rated as good or okay by the majority of women	Poor
Watson-Jones 2012 [81]	Compare coverage achieved by two different delivery strategies (class-based vs. age-based) for HPV vaccine among schoolgirls	Tanzania	HPV vaccine	Educate: community outreach with lectures, pamphlets, posters, radio messages, and dramas Restructure: change delivery models class-based vs. age-based	Acceptability: reasons for refusal Penetration: HPV vaccine coverage and 3 dose adherence	For each dose, coverage was higher in class-based schools than in age-based schools (dose 1: 86.4 vs 82.0% [*p* = .30]; dose 2: 83.8 vs 77.8% [*p* = .05]; and dose 3: 78.7 vs 72.1% [*p* = .04]).	Poor
Nonrandomized control trials							
Mutyaba 2009 [83]	Evaluate the efficacy of male partner involvement in reducing loss to follow-up among women in Uganda referred for colposcopy after a positive cervical cancer screening test	Uganda	VIA,VILI, colposcopy	Educate: group lecture, incentivize follow-up with inclusion of male partner by sending educational pamphlet home for partners	Penetration: screening coverage, loss to follow-up	Intervention group was significantly more likely to return for colposcopy than the control group, with 16 and 34%, respectively, lost to follow-up.	Poor
Pre-post tests							
Abiodun 2014 [67]	Determine the effect of health education on the awareness, knowledge and perception of cervical cancer and screening among women in rural communities	Nigeria	Unspecified screening	Educate: 1-day health education intervention with group didactic lectures and an educational movie	Penetration: screening coverage	There was a statistically significant difference in cervical cancer awareness, perception, knowledge and screening uptake between intervention and control groups. Proportion of women in the intervention group who had undertaken screening rose from 4.3 to 8.3% (*p* = .038).	Good
Adamu 2012 [70]	Assess the effect of health education on the knowledge, attitude, and uptake of Pap smear among female teachers	Nigeria	Pap smear	Educate: individual counseling on cervical cancer, complications, cost, importance of screening Finance: free coupon for pap test	Penetration: Pap smear screening coverage	The proportion of respondents with a reported practice of Pap smear was low and similar in both groups (1.1 in the intervention group and 4.9% in the control group, *p* = 0.16). Uptake was poor at post-intervention phase for both groups (*p* = .45).	Good
Caster 2017 [71]	Assess the acceptability, feasibility and effectiveness of a tablet-based cervical cancer educational intervention	Malawi	Unspecified screening	Educate: 30-min tablet-based education	Acceptability: participants’ preference for tablet vs. in-person education Feasibility: ease of tablet use, number of times participants need assistance with tablet Adoption: intention to screen	The median pretest score was 11 out of 20 and the median posttest score was 18 (*p* < 0.001). 226 participants (93%) stated that they would like to obtain cervical cancer screening	Fair
Chigbu 2017 [72]	Determine the impact of trained community health educators on the uptake of cervical and breast cancer screening and HPV vaccine in rural communities	Nigeria	HPV vaccine	Educate: house-to-house education given on a one-on-one basis by community health workers on cervical and breast cancer prevention	Penetration: screening and HPV vaccination coverage	Of the 1327 enrolled women, 42 (3.2%) had undergone screening pre-intervention and 897 (67.6%) received screening afterwards (*p* < .0001). Only 2 (0.9%) of 214 children eligible for HPV vaccination had received the vaccine before versus 71 (33.2%) after the intervention (*p* < .001).	Fair
De Groot 2017 [73]	Provide information on STI knowledge and vaccine acceptance after an educational session	Mali	HPV vaccine	Educate: educational session to inform adults and adolescents about HPV and cervical cancer, symptoms and causes, benefits and availability of the HPV vaccine	Adoption: parent and child reported willingness to accept the HPV vaccine	The education session increased the HPV vaccine acceptance in all groups, especially among adolescents (from 75.3 to 91.8%, *p* < .01).	Fair
Dreyer 2015 [68]	Measure changes in knowledge and screening behavior after an educational intervention provided to mothers of adolescent HPV vaccine recipients	South Africa	Pap smear, HPV DNA test	Educate: 15 min didactic lecture and educational pamphlets Restructure: integrate screening of mothers into child HPV vaccination program	Penetration: screening coverage	Knowledge about symptoms (*p* < .005), screening (*p* < .005), and vaccination (*p* < .05) improved significantly at 6 month retesting. Improvement for reported screening in the past 12 months was more favorable in Gauteng (41%) with self-sample than in Western Cape with Pap smear (26%).	Fair
Levine 2011 [75]	Determine the effectiveness of an educational program in VIA knowledge and skills retention among healthcare providers in 2 countries	Uganda	VIA	Educate: 5 day educational program for providers with didactic lectures and procedural training in VIA	Acceptability: provider reported comfort with skills Sustainability: skill assessment at 6-month follow-up	Mean test scores increased significantly after participation in the training session (62% vs. 81%, *p* < .001). Self-reported comfort level for identifying cellular abnormalities also increased (2.1 vs. 3.3; *p* < 0.001 There was no significant difference between initial and 6-month follow-up test scores (80 vs. 79%).	Poor
Mbachu 2017 [74]	Assess the effectiveness of peer health education on perception, willingness to screen and uptake of cervical cancer screening of women during Anglican church meetings	Nigeria	Pap Smear, VIA, VILI	Educate: three 45–60 min sessions repeated monthly of peer health education on cervical cancer burden, risk factors, symptoms and prevention	Penetration: Pap smear and VIA/ VILI screening coverage Adoption: willingness to screen	Screening rate increased by 6.8% and the observed difference was statistically significant (*p* = 0.02).	Fair
Miller 2007 [76]	Evaluate a train the trainer program for cervical screening implementation and assess pre-post knowledge of the implementation process	Nigeria	VIA, VILI, Pap smear, Cryotherapy	Educate: train the trainer in implementation	None	Of the 41 evaluable exams, 9 saw no change, 31 showed improvement, 1 scored worse.	Poor
Wright 2010 [69]	Evaluate the effect of a health education program on knowledge of cervical cancer among market women in an urban area	Nigeria	Pap smear	Educate: develop pamphlets, community outreach	None	Significant increase in proportions were found in the intervention/experimental group on awareness of cervical cancer (61.7%), associated symptoms and risk factors such as early sexual debut, promiscuity and smoking.	Fair
Cross-sectional studies							
Adamson 2015 [42]	Determine the acceptability and accuracy of tampon-based self-collection for hrHPV mRNA testing in HIV-infected women	South Africa	HPV RNA test	Restructure: self- vs. physician-HPV RNA sampling	Acceptability: care, privacy, embarrassment, discomfort, pain, preference Fidelity: concordance between physician- and self- collected samples	There was no difference in test positivity between clinician-collection, 36.7%, and tampon- collection, 43.5% (*p* value = 0.08). Using clinician-collection as the reference, the sensitivity and specificity for hrHPV mRNA of tampon-collection were 77.4 and 77.8%, respectively.	Good
Adepoju 2016 [59]	Determine sociodemographic characteristics, awareness and uptake of a free cancer screening program	Nigeria	Pap smear	Educate: public sensitization with women groups and mass media campaign Finance: free screening	Penetration: Pap smear screening coverage	287 women were screened but uptake of cervical cancer screening was low since most women did not come for the program despite the public sensitization.	Poor
Asgary 2016 [22]	Evaluate the feasibility and efficacy of ongoing, smartphone-based support in sustaining VIA skills for community health nurses	Ghana	VIA, digital imaging	Educate: 2-week didactic and procedural training for VIA and digital imaging, ongoing consultation Quality: audit and feedback for digital images via smartphone messaging within 24 h	Fidelity: inter-rater agreement for VIA between nurses and expert physician Feasibility: VIA picture quality	Agreement rate between all VIA diagnoses made by all CHNs and the expert reviewer was 95%. Cohen κ statistic was 0.67 (95% CI = 0.45**–**0.88). Images for 9 patients, taken by 6 CHNs, were unclear.	Fair
Awua 2017 [49]	Compare the uptake of screening between a community-based vs. hospital-based strategies for collecting HPV DNA samples	Ghana	HPV DNA test	Educate: community lectures at churches Restructure: community-based vs. hospital-based specimen collection Quality: patient phone reminders	Penetration: HPV DNA testing coverage	Response rates were higher for community-based (95.1%) than short-term (46.6%) or long-term (38.5%) hospital-based appointments	Fair
Catarino 2015 [48]	Evaluate the use of smartphone telemedicine for off-site diagnosis of cervical intraepithelial neoplasia	Madagascar	VIA, VILI, HPV DNA test, Digital imaging	Restructure: on-site vs. off-site evaluation of VIA digital images	Fidelity: sensitivity and specificity between on-site physician diagnosis and off-site assessment via digital images	The on-site physician had a sensitivity of 66.7% and a specificity of 85.7%; the off-site physician consensus sensitivity was 66.7% with a specificity of 82.3%.	Good
Crofts 2015 [61]	Report on women’s acceptance of HPV self-sampling following an education intervention on cervical cancer and HPV	Cameroon	HPV DNA test	Educate: 20 min didactic lecture and educational pamphlet with instructions for HPV self-samplings	Acceptability: embarrassment, pain, anxiety, confidence, discomfort, relaxation, complexity	Overall, participants showed high acceptability scores for HPV self-testing (6.986 of 24), with lower scores being more favorable. However, there was no difference in acceptability between participants with good vs. poor knowledge scores.	Fair
DeGregorio 2017 [57]	Evaluate a nurse-led, fee-for-service cervical cancer screening program using visual inspection with acetic acid-enhanced by digital cervicography in the setting of a large faith-based health care system	Cameroon	VIA, VILI, Digital imaging, Cryotherapy, LEEP, Biopsy	Quality: quarterly meeting to review cervicographs with expert clinician Educate: peer educators with group lectures in the community Finance: fee-for-service sliding scale based on community demographics Restructure: integrate with family planning, breast exams, STI testing	Penetration: VIA screening coverage	In 8 years, 44,979 women were screened for cervical cancer.	Poor
Dim 2015 [62]	Assess willingness to pay out-of-pocket for Pap smear among HIV positive women after provided information about cervical cancer and screening	Nigeria	Pap smear	Educate: individual counseling on increased risk for cervical cancer, Pap smear protocol, and costs	Adoption: willingness to pay for Pap smear	378 (94.5%) respondents were willing to pay for Pap smear, irrespective of the cost. Willingness to pay showed no trend across age groups (*p* = . 148), marital status groups (*p* = . 890), educational status groups (*p* = . 337), and parity groups (*p* = . .611).	Fair
Firnhaber 2015 [41]	Determine whether a quality assurance program using digital cervicography improved the performance of VIA to detect cervical intraepithelial neoplasia grade 2 or worse (CIN 2+) in HIV-infected women	South Africa	VIA, digital imaging	Educate: 2-week VIA training Quality: audit and feedback of VIA cervical images by expert gynecologist in weekly QA meetings	Fidelity: sensitivity and specificity of VIA compared between nurses’ visual assessment and physician digital image assessment	There was substantial agreement between the VIA real-time readings of the nurse and that of the physician with digital cervicography (*k* statistic = 0.69). There was no statistical difference between the ability of nurses to detect CIN 2+ at the beginning and at the end of the study.	Poor
Goldhaber-Fiebert 2009 [65]	Determine the relationship between investment in community health worker (CHW) home visits and increased attendance at cervical cancer screening appointments	South Africa	Unspecified screening, colposcopy, biopsy	Quality: patient reminder system with community health worker (CHW) home visits to encourage attendance to follow-up appointments	Costs: total CHW program cost, average cost per women screened Penetration: screening coverage, total CHW home visits completed, patient adherence to appointments	Adherence increased from 74 to 90%; 55 to 87%; 48 to 77%; and 56 to 80% for 6-, 12-, 24-, and 36-month appointments. The CHW program cost R194,018 with 1576 additional appointments attended. Average per-woman costs increased by R14–R47.	Good
Horo 2012 [23]	Determine effect of a phone based tracking system on follow-up rates	Cote d’Ivoire	VIA, VILI, colposcopy, biopsy	Educate: individual counseling, group patient teaching, and educational pamphlets Quality: phone based patient reminder system with maximum of 3 calls (one per week)	Acceptability: patient reasons for loss to follow-up Penetration: colposcopy loss to follow-up rates	The use of a phone-based tracking enabled a significant reduction of women not attending medical consultation after initial positive screening from 36.5 to 19.8% (*p* < 10–4). Reasons for not following up include cost, transportation, fear and time	Poor
Huchko 2011 [50]	Assess the impact of a cervical cancer screening prevention pilot project implemented into an established AIDS program	Kenya	VIA, colposcopy, biopsy, LEEP	Educate: 1-week training for providers in VIA, colposcopy, and lab specimen processing, individual patient counseling and community outreach Restructure: increase lab capacity, embed in HIV program Quality: ongoing for consultation for program protocol through CCSP	Acceptability: reasons for patients refusing screening, provider satisfaction with training and program implementation Penetration: VIA screening coverage, provider training coverage Feasibility: challenges to implementing the program	High coverage (87%). Reasons for declining screening included partner support, menstruation, and fear. 28 (90%) clinical officers underwent training in VIA and colposcopy. The main challenges reported were related to infrastructure limitations (lack of water, electricity and supplies; and long waits in the clinic) and perceived patient barriers	Poor
Kapambwe 2013 [60]	To evaluate knowledge transfer after training of traditional marriage counselors (alangizi) to integrate cervical cancer lessons into their routine counseling	Zambia	VIA, Digital imaging	Plan: develop trust between alangizi and research team Educate: one-day training on basic cervical cancer knowledge for traditional marriage counselors Restructure: integrate cervical cancer messaging into marriage counseling	Feasibility: perceived barriers and facilitators of integrating screening	A majority of the trainees correctly associated cervical cancer with HPV (35.6%) and multiple sexual partnerships (28.9%).	Poor
Khozaim 2014 [51]	Determine the challenges and successes of integrating a public-sector cervical screening program into a large HIV care system	Kenya	VIA, VILI, digital imaging, colposcopy, biopsy, cryotherapy, LEEP	Educate: community outreach, mass media Restructure: embed in HIV care Quality: patient reminder system with calls and text messages for upcoming appointments	Penetration: loss to follow-up rates	31.5% lost to follow-up (27.9% colposcopy to biopsy, 49.3% biopsy to LEEP, 59.6% colposcopy to chemo or hysterectomy)	Poor
Lack 2005 [31]	Compare two self-administered techniques for detecting HPV (tampons and swabs) with a clinician directed technique (cervical cytobrush)	Gambia	HPV DNA test	Restructure: self-administration- vs. physician-collected swabs	Fidelity: sensitivity and specificity compared between self- and physician collected cervical swabs Penetration: screening coverage	Self-administered swabs showed a sensitivity of 63.9% and tampons showed a sensitivity of 72.4% compared to the cervical cytobrush as the gold standard. The acceptability of these two tests was 97.1 and 84.6%, respectively.	Poor
Ladner 2012 [44]	Assess the effectiveness of school vs. clinic based delivery models on HPV vaccine coverage in 7 different countries	Cameroon	HPV vaccine	Restructure: change service sites of HPV vaccination (school, clinic, and mixed models)	Penetration: Vaccine coverage and adherence	High coverage (88%) and adherence (91%) across programs. Mixed model in both school and clinic settings was most effective.	Fair
LaMontagne 2011 [45]	Assess the effectiveness of school vs. clinic based delivery models on HPV vaccine coverage in 4 different countries	Uganda	HPV vaccine	Educate: community outreach and educational pamphlets Restructure: change service sites of HPV vaccination (health center, school, and integrated with other health program)	Acceptability: reasons for vaccine acceptance or refusal Penetration: HPV vaccination coverage	High school coverage (88.9%) but low health center coverage. Reasons for accepting the HPV vaccine that: (i) it protects against cervical cancer; (ii) it prevents disease, or (iii) vaccines are good. Refusal was more often driven by programmatic considerations (e.g., school absenteeism) than by opposition to the vaccine.	Poor
Maree 2012 [53]	Determine whether cervical screening uptake could be improved when breast and cervical screening are combined	South Africa	VIA	Educate: one-on-one patient counseling Restructure: combine cervical cancer and breast cancer screenings	Acceptability: patient reasons for screening refusal Penetration: VIA screening coverage	Moderate coverage (65.4). Major reason for refusal was menstruation.	Good
Megevand 1996 [46]	Determine the feasibility of providing a cervical screening facility to the underprivileged communities through an educational program and mobile clinic	South Africa	Pap smear, colposcopy, LEEP	Educate: community outreach Restructure: change service site to mobile clinic with same day Pap smear results and treatment if indicated Quality: audit and feedback for 100 of every 300 cytology slides	Penetration: loss to follow-up rates	Loss to follow-up rates were much lower for minimal delay, mobile delivery (3%) compared to longer delay, clinic delivery (66%)	Poor
Mehotra 2014 [58]	Assess the impact of enrollment in an incentive program on receipt of eight preventive care services including Pap smear	South Africa	Pap smear	Finance: insurance incentive program	Penetration: Pap smear screening coverage	65.5% (2,742,268) of health plan members enrolled in the incentive program at some point. Odds ratio for receipt of Pap test is 2.17	Good
Michelow 2006 [66]	Determine if rapid review of reportedly negative cervical smears is a useful internal quality assurance modality in an unscreened population with very high rates of cervical carcinoma	South Africa	Pap smear	Quality: quality monitoring system for randomly selected Pap smear slides by a senior cytotechnologist	Fidelity: sensitivity and specificity	An amended report was sent out in 373 (0.59%) of the 62,866 cervical smears. The false-negative proportion for HSIL and ASC-H (combined) in this study was 5.76%.	Fair
Moodley 2013 [52]	Demonstrate the capacity of school health teams to carry out vaccinations within a school environment	South Africa	HPV vaccine	Restructure: integrated with cervical cancer screening program for mothers Educate: staff training in program policy, sensitize school leadership, community outreach	Penetration: HPV vaccine coverage and 3 dose adherence	High coverage and adherence of the vaccine was found to be high: 99.7, 97.9, and 97.8% for the first, second, and third doses, respectively.	Poor
Moon 2013 [54]	Assess the feasibility, successes and challenges of integrating a VIA program into an existing HIV program	Mozambique	VIA, Cryotherapy, LEEP, Colposcopy	Educate: 1-week didactic and procedural training in VIA and cryotherapy Restructure: change service sites—embed in HIV care	Feasibility: reasons for delay in treatment provision Penetration: cryotherapy and LEEP follow-up rates Sustainability: percentage of providers still performing VIA in 1 year	High and improved follow-up rates between first (53%) and the last quarter (96%) cryotherapy same day coverage rates. High (88%) referral follow-up rates. 0% physicians and 50% nurses continued VIA screening 1 year after training. Delays in treatment include equipment theft and malfunction.	Poor
Obiri-Yeboah 2017 [43]	Determine the acceptability, feasibility and performance of alternative self-collected vaginal samples for HPV detection	Ghana	HPV DNA test	Restructure: self- vs. physician-HPV DNA sampling	Acceptability: ease of use, preference Fidelity: concordance between physician- and self- collected samples	The overall HPV detection concordance was 94.2% and kappa value of 0.88 (*p* < 0. 0001), showing excellent agreement. 57.7% preferred self- to physician collection.	Fair
Ogembo 2014 [47]	Inform the Cameroon Ministry of Health of the acceptability, feasibility, and optimal delivery strategies for HPV vaccine	Cameroon	HPV vaccine	Educate: community awareness campaign using mass media, pamphlets, and posters Restructure: change delivery sites (clinic, school, community/mobile), integrate with screening of mothers Quality: patient reminder system with peer tracking (school)	Feasibility: vaccines lost/damaged/expired, adverse events Penetration: vaccine coverage, refusal rate, 3 dose adherence	Total of 6851, 6517 and 5796 girls were immunized with the first, second and third doses of HPV vaccine, respectively, achieving 84.6% full dosage coverage of the adolescents who received the first dose. Only 63 of the 19,200 doses received were lost, damaged or expired. CBCHS charged a fee of US$8 per 3-dose series only to those who were able to pay. Despite the fee, 84.6% of the 6851 girls who received the first dose received all three doses.	Poor
Quinley 2011 [25]	Examine the diagnostic agreement between off-site expert diagnosis using photographs of the cervix (photographic inspection with acetic acid, PIA) and in-person VIA	Botswana	HPV DNA test, VIA, digital imaging	Quality: quality assurance for digital cervical images	Feasibility: rate of equipment malfunction Fidelity: inter-rater reliability with expert, concordance between VIA and PIA	Moderate to high agreement (69–100%) with expert, varied for each nurse High concordance (70%) between PIA and VIA results 31 images were insufficient for reading.	Fair
Ramogola-Masire 2012 [24]	Determine the feasibility and efficiency of the **“**see and treat**”** approach using visual inspection acetic acid (VIA) and enhanced digital imaging (EDI) for cervical cancer prevention in HIV-infected women	Botswana	VIA, VILI, cryotherapy, digital imaging	Educate: 3-day didactic teaching and 8 weeks of procedural training in VIA, digital imaging, and cryotherapy Restructure: embed in HIV care Quality: audit and feedback of cervical images by expert gynecologist in weekly quality control meetings	Fidelity: sensitivity, specificity, inter-rater reliability of VIA assessments between nurses and expert gynecologist Penetration: cryotherapy follow-up rates	High agreement between nurses and the gynecologist in the evaluation of digital pictures (83.3%) Overall follow-up 709 of 842 (84.2%)	Fair
Safaeian 2007 [28]	Compare human papillomavirus (HPV) DNA testing between self-administered vaginal swabs and physician-administered cervical swabs	Uganda	HPV DNA test	Restructure: self-administration vs. physician collected swabs	Fidelity: sensitivity and specificity between self- and physician- collected samples Penetration: screening coverage	Compliance with self-collected swabs was > 86%; however, only 51% accepted a pelvic examination. Agreement among paired observations was 92% with a kappa statistic of 0.75.	Good
Synman 2015 [55]	Investigate the feasibility of linking HPV self-testing for mothers with a two-dose HPV vaccination schedule of their daughters	South Africa	HPV DNA test, HPV vaccine	Educate: educational pamphlets sent home with children for mothers Restructure: integrate HPV DNA self-sample kit for mothers into vaccination program for daughters	Penetration: HPV DNA self-testing coverage	Of the 1135 self-screen kits handed out to eligible girls to be passed on to their female guardians, 160 women participated in the self-screening (14.1%).	Poor
Ting 2013 [27]	Compare the performance of hrHPV mRNA testing of physician- and self-collected specimens for detecting cytological high-grade squamous intraepithelial lesions or more severe (QHSIL) and examined risk factors for hrHPV mRNA positivity in female sex workers	Kenya	HPV RNA test, Pap smear	Restructure: self-administration vs. physician-collected swabs	Fidelity: sensitivity and specificity compared between self- and physician collected cervical swabs	Overall sensitivity of hrHPV testing for detecting QHSIL was similar in physician-collected (86%) and self-collected specimens (79%). Overall specificity of hrHPV mRNA for QHSIL was similar in both physician-collected (73%) and self-collected (75%) specimens.	Good
Tum 2013 [64]	Determine if a community health worker and education intervention could increase screening uptake	South Africa	Unspecified screening	Educate: health worker training, community education	Acceptability: patient perceived value of community health worker Penetration: screening coverage	Low coverage (3%). All found value in health worker through informing, teaching, and motivating.	Fair
Untiet 2014 [30]	Test differences in performance between self-HPV versus physician-HPV and their ability to detect abnormal cytology results	Cameroon	HPV DNA test	Restructure: self-administration vs. physician collected swabs	Fidelity: sensitivity and specificity compared between self- and physician collected cervical swabs	HPV prevalence was 14.6 and 12.7% for self-HPV and physician-HPV, respectively (Cohen’s kappa = 0.74). HPV positivity by cytological diagnosis for ASC-US+ was similar with the two tests	Good
Wamai 2012 [63]	Evaluate the effectiveness of a campaign in sensitizing parents to HPV vaccination and influencing uptake of vaccine for their children	Cameroon	HPV vaccine, VIA, digital imaging	Educate: Community outreach, mass media, education program	Acceptability: reasons to vaccinate or not Adoption: willingness to vaccinate Penetration: VIA screening coverage, sensitization campaign coverage	High willingness to vaccinate among parents. Low coverage (35.3%) of VIA screening among parents. Low education program coverage with 5.9% surveyed parents learning about cervical cancer from program. Top reasons not to vaccinate include effectiveness (31.8%), safety (18.4%), provider recommendations (17.8%) and cost (16.6%).	Fair
Were 2010 [56]	Pilot test and assess the feasibility of integrating VIA screening into an existing maternal child health and family planning program	Kenya	VIA, VILI	Educate: VIA/VILI training Restructure: change service- embed in maternal child health and family planning	Penetration: VIA/VILI screening coverage and loss to follow-up	Moderate coverage and follow-up. 435 invited—216 declined 219 accepted. 24 of 40 went for colposcopy.	Poor

### Study design

The majority of studies included in the review are cross-sectional (*n* = 34, 64.2%). Ten of the cross-sectional studies similarly evaluated the impact of changing service providers on how well the screening test is performed. Using specificity and sensitivity rates, some studies compared VIA assessments between nurses and an expert physician [22, 24, 25, 41] while others compared self- vs. physician-collected samples for HPV DNA testing [27, 28, 30, 31, 42, 43]. Sixteen studies examined if screening coverage increases when changing service sites [44–49], combining screening with an already established program (i.e., HIV/STI screening) [50–57], or providing financial incentives [58, 59]. Four studies evaluated the effect of educational interventions on knowledge, attitudes, and screening behaviors for patients [60] and providers [61–63]. Three studies examined if reminder systems can help to decrease lost to follow-up rates through community health workers [64, 65] or phone-based tracking [23]. One study, Michelow et al. [66], used rapid review of reportedly negative cervical smears as an internal quality assurance modality.

Ten studies (18.9%) were conducted with a pre-posttest design. All of the pre-post studies evaluated the effectiveness of educational interventions in improving awareness and screening behaviors for patients [67–74] or knowledge and skills retention for providers [75, 76]. Only three studies included a control group [67, 69, 70].

There are eight randomized control trials (15.1%). Six trials tested strategies to increase screening uptake through educational interventions [26, 77–79], financial incentivizes [80], or changing service sites [81]. Two trials compared HPV DNA self-sampling to the current standard of physician collection via speculum exam [29, 82].

Only one study is a non-randomized control trial (1.9%). Mutyaba et al. [83] evaluated if male partner involvement is effective in reducing loss to follow-up after a positive VIA screening test.

### Prevention tools

Primary prevention with HPV vaccine was included in 9 studies (17.0%). VIA was the most frequently used secondary screening method (*n* = 19, 35.8%). Less commonly, secondary screening was completed with HPV DNA/mRNA testing (*n* = 15, 28.3%), Pap smear (*n* = 13, 24.5%), VILI (*n* = 9, 17.0%), colposcopy (*n* = 7, 13.2%), biopsy (*n* = 5, 9.4%), and unspecified screening (*n* = 5, 9.4%). Digital imaging to supplement visual screening methods (VIA/VILI) was used in 9 studies (17.0%). If follow-up treatment of precancerous lesions was conducted, it was either performed with LEEP (*n* = 5, 9.4%) or cryotherapy (*n* = 5, 9.4%).

### Implementation strategies

Researchers used educate (*n* = 38, 71.7%), restructure (*n* = 26, 49.1%), and quality (*n* = 13, 24.5%) strategies most frequently in their studies. For patients and their families, education strategies aimed to increase cervical cancer awareness and the importance of prevention. For providers, education strategies were used to improve knowledge and skills retention in conducting screening and treatment services such as VIA, cryotherapy, and LEEP. Example educate strategies include community outreach, individual patient teaching and counseling, provider training, mass media campaigns, and development of educational materials. Restructure strategies were used to facilitate implementation by changing service sites (established vs. mobile clinic for Pap smear), changing delivery models (age- vs. class-based for HPV vaccine), or changing providers (nurse vs. physician for VIA, patient vs. physician for HPV DNA test). Several studies also used the restructure strategy to combine cervical cancer prevention with other services (i.e., HIV/STI testing, marriage counseling, family planning) to improve the financial and infrastructural support provided through already established programs. The quality strategies included in these studies were ongoing consultation, patient reminder systems, and audit-feedback mechanisms. Five studies (9.4%) included a finance strategy to incentivize patients to uptake screening services. Only one study (1.9%) utilized the plan strategy. Kapambwe et al. [60] spent time developing trust with alangizi (traditional marriage counselors) to encourage them to integrate cervical cancer screening messaging into their counseling sessions with women. There were no policy strategies (0%) in the included studies.

### Implementation outcomes

The most studied implementation outcomes were penetration (*n* = 33, 62.3%), acceptability (*n* = 15, 28.3%), and fidelity (*n* = 14, 26.4%). Penetration was often measured as vaccine or screening coverage, which is calculated by dividing the number of women who participated by the total eligible or targeted population. Additional measures of penetration included rates of loss to follow-up for cryotherapy or LEEP treatment and three-dose adherence for HPV vaccination. Acceptability was most commonly measured by surveying patients to determine reasons why they accepted or refused participation. Among providers, acceptability was measured as comfort with performing newly learned skills and reported satisfaction with training and program implementation. Fidelity was measured in studies that compared either nurses’ VIA assessments or self-collected HPV DNA samples to that of expert physicians. These comparisons indicated whether patients and nurses could perform these tests with reasonable reliability and help to address physician shortages by alternatively implementing the screenings.

Other less frequently studied outcomes included feasibility (*n* = 8, 15.1%), adoption (*n* = 6, 11.3%), sustainability (*n* = 2, 3.8%), and cost (*n* = 1, 1.9%). To measure feasibility, many researchers determined providers’ perceived barriers and facilitators to implementation. Other studies quantified circumstances that impeded successful operation of the program such as rates of equipment malfunction, poor picture quality for digital images, invalid lab results, and expired vaccines. Adoption was measured as the willingness or intent of patients to participate in screening or HPV vaccination. Only two studies included measures of sustainability. Moon et al. [54] quantified sustainability by the number of providers that were still performing VIA 1 year after initial training. Levine et al. [75] determined VIA skill and knowledge retention with a 6-month follow-up assessment. One study, Goldhaber-Fiebert et al. [65], measured costs associated with cervical cancer screening, i.e., community health worker home visits.

There were no studies that measured appropriateness (0).

### Quality assessment

Few studies (*n* = 11, 20.8%) were determined to be of “good” quality using the NHLBI Quality Assessment Tools. The remaining studies were “fair” (*n* = 22, 41.5%) or “poor” (*n* = 20, 37.7%). Overall, many studies did not sufficiently describe their methodology, which made it difficult to make determinations for items on the NHLBI tools. Items were often marked as “not specified” or “cannot be determined.” A common weakness specifically for controlled intervention studies was a lack of adequate randomization. Some randomized control trials (RCTs) used a preset plan for allocating patients to intervention or control groups (i.e., even vs. odd ID numbers) instead of using computer-generated lists. Other RCTs did not provide any description for how participants were allocated. Adequate randomization is important as it provides confidence that results are attributable to the intervention rather than a difference in groups at baseline. For pre-posttests, only 3 of the 10 studies included a control group [67, 69, 70]. Without a control group for comparison, there is less confidence that an improvement between pre- and post-assessments is due to the intervention rather than mere chance. The cross-sectional studies were mainly descriptive. Limited cross-sectional studies used statistical analyses to determine associations between intervention and outcomes. Confounders were rarely measured and included in the analyses. Outcome measures frequently lacked validity and reliability.

## Discussion

The challenges of establishing and sustaining cervical cancer prevention programs in SSA have been identified in several recent reviews [4–7]. However, the authors have found no review to date that addresses implementation strategies to overcome these identified barriers. Safe and effective prevention tools exist but are not reaching the women that need these services most. This review is an attempt to enter cervical cancer prevention into the implementation science conversation to propel the state of the science forward. Finocchario-Kessler et al. [84] conducted a systematic review of the literature between 2004 and 2014 to characterize the cervical cancer research in SSA according to four public health categories (primary prevention, secondary prevention, tertiary prevention, and quality of life). They determined that most studies focused on secondary prevention and concluded that there is a need for “implementation science research to inform feasible and sustainable strategies to maximize the number of women reached with services” [84].

Implementation science is an emerging field that aims to bridge research and practice in order to ultimately achieve desired patient and population health outcomes [85]. Historically, a significant amount of efficacy and effectiveness research conducted in controlled settings has not translated into “real-world” impact. The traditional, passive methods of dissemination (i.e., journal publishing) have not proven effective. Estimates show that it takes an average of 17 years for 14% of original research to effect practice [86]. Implementation science seeks to address this “quality chasm” by explicitly studying the processes of implementing evidence-based programs in clinical and public health settings [87]. Implementation strategies are instrumental in bridging the gap and improving the speed and rigor of research translation. The results from this review have provided insight into how study design, strategies, and outcomes have been used to study implementation of cervical cancer prevention in SSA. Since sub-Saharan Africa faces some of the highest cervical cancer rates worldwide, it is important to evaluate what has been done so far to address these challenges and contemplate how these efforts can be improved through use of implementation strategies.

### Study design

While randomized control trials are the “gold standard” in efficacy and effectiveness research, these study designs are difficult to feasibly conduct in implementation research due to the use of multi-level, multi-strategy interventions [85]. It is more difficult to conduct random assignment when the level of analysis is at the organization, community, and/or country level rather than the individual level. It is also difficult to produce large enough sample sizes to create adequate statistical power. For these reasons, Brownson et al. [85] conclude in *Dissemination and Implementation Research in Health*, one of the seminal works to progress the field of implementation science, that quasi-experimental designs without randomization are reasonable for implementation research. However, they argue that rigorous quasi-experimental design is essential to achieving quality data that has practical use. While quasi-experimental studies may be more feasible to conduct, these designs do not produce the same level of confidence in causation as randomized control trials and make it more difficult to compare effectiveness between different studies.

In the absence of randomization, researchers can incorporate control groups, confounders, and statistical comparison of baseline group characteristics to greatly increase rigor of implementation study designs. In their assessment of 66 Cochrane reviews on implementation research, Brownson et al. [85] concluded that “many publications in the literature are still merely descriptive in nature or have weak designs without comparison or control conditions to answer critical research questions.” This systematic review has produced similar results. The majority of studies are cross-sectional, descriptive studies and assessed as “poor” or "fair" quality. This review echoes the argument that there is a need for more rigorous research designs that meet the needs of implementation science questions.

### Implementation strategies

Evaluating effectiveness for the various implementation strategies is difficult due to the descriptive nature of most studies, overall poor quality in study designs, and variation in outcomes measured. While educate strategies were the most popular method leveraged in attempt to improve implementation, implementation science suggests that dissemination of information is not the most effective method for creating sustainable change [88]. Within this literature review, education has also failed to produce intended outcomes. Many studies employing educate strategies have shown improvements in awareness. However, these strategies in isolation have not always catalyzed better uptake, acceptability, and/or confidence [61, 63, 64, 78]. If a significant difference was observed, uptake still remained low [67, 77, 83]. These results suggest a need to diversify implementation strategies used to improve cervical cancer prevention in this context. Restructure, finance, and attend to policy context strategies can provide the organizational support required to improve implementation and overcome barriers particular to resource-limited settings.

### Implementation outcomes

While there were implementation outcomes included in these studies, the overwhelming majority were patient-level outcomes, such as symptomatology, cancer rates, cervical lesion typology, etc. For implementation studies, it is crucial to measure implementation outcomes specifically [37]. If the desired health outcomes are not achieved after an evidence-based program is implemented, the failure is typically attributed to the evidence-based program without consideration of how well the practice was or was not implemented in that particular setting [86, 88]. If we do not measure implementation outcomes, there is no way to deduce what is ultimately influencing the patient or population health outcomes. Additionally, there is a need for continued effort in operationalizing and measuring implementation outcomes. One of the eight outcomes (appropriateness) was not measured in the review and should be considered for inclusion in future studies.

### Limitations

A major limitation of this systematic review is the overall quality of evidence. “Poor” and "fair" quality ratings for the majority of studies make it difficult to make conclusions about implementation strategies and their effectiveness. Risk of bias in the study design and implementation greatly decreases confidence in the validity of results. Another limitation is that only a sample of initial articles, rather than the entire dataset, were abstracted and quality assessed by a second reviewer. However, inconsistencies were resolved through consensus before the primary reviewer proceeded with the remaining articles to ensure accuracy.

## Conclusions

This systematic review elicits the need to diversify strategies that are used to improve implementation for cervical cancer prevention programs. While education is important, implementation science literature reveals that dissemination of information in isolation is not as effective in generating change [88]. There is a need for additional organizational support to further incentivize and sustain change [85, 89]. Implementation research is difficult because interventions are multifaceted and conducted at different levels of analysis [85]. Many studies in this review included patient level outcomes but did not include implementation-specific outcomes to assess the success of implementation strategies. This review calls for an increased use of implementation science frameworks to inform the design of studies that aim to improve cervical cancer prevention in SSA. This review also calls for increased use of common terminology from implementation science for outcomes and strategies. Implementation science can help to communicate results between researchers and increase rigor of research design to better isolate impact of implementation strategies on intended outcomes.

## Additional file

Additional file 1: Database-Specific Search Strategies. (DOCX 2827 kb)

## Abbreviations

HPV: Human papillomavirus
LEEP: Loop electrosurgical excision procedure
NHLBI: National Heart, Lung, and Blood Institute
PRISMA: Preferred Reporting Items for Systematic Reviews and Meta-Analyses
RCT: Randomized control trial
SSA: Sub-Saharan Africa
VIA: Visual inspection with acetic acid
VILI: Visual inspection with Lugol’s iodine
