# Supplementary figures and images for: Implementation strategies to improve cervical cancer prevention in sub-Saharan Africa: a systematic review
Source: Implement Sci. 2018 Feb 9;13:28. doi: 10.1186/s13012-018-0718-9 (PMC5807829; doi:10.1186/s13012-018-0718-9)

Pub Med


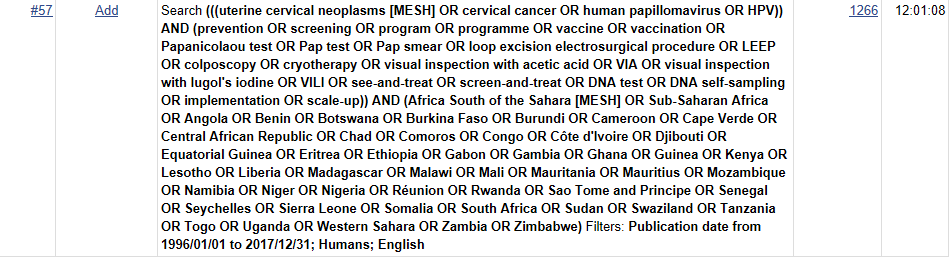


Web of Science

**
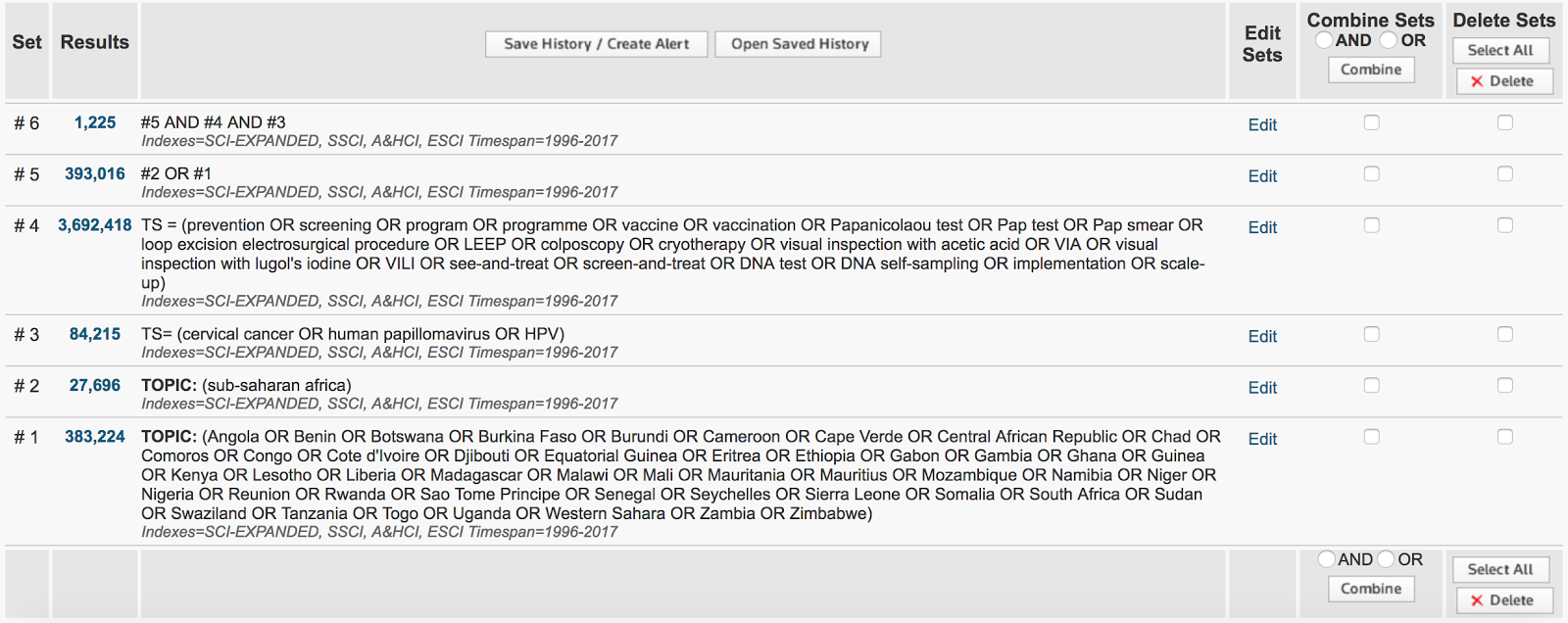
**

Ovid/Medline


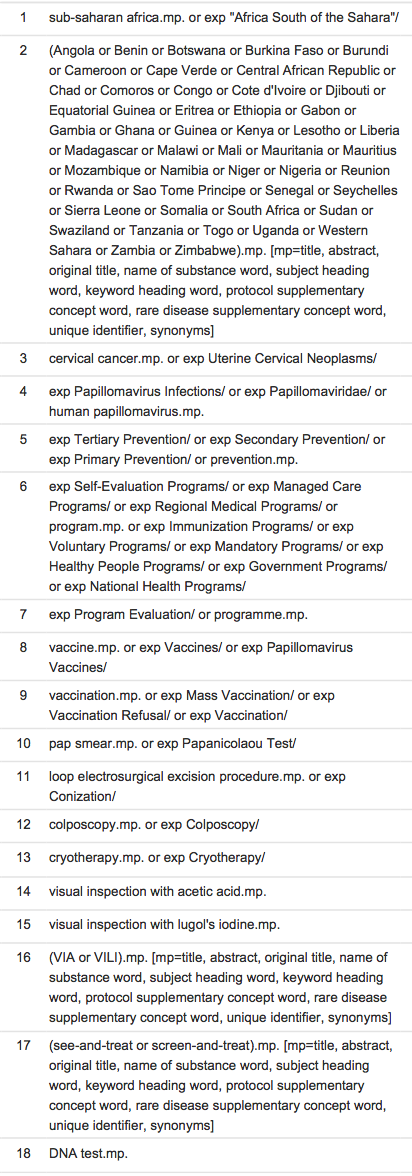


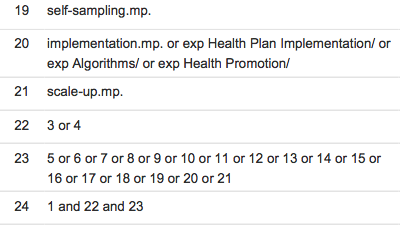

Supplement: Supplementary file 1 — Database-Specific Search Strategies. (DOCX 2827 kb) [file 13012_2018_718_MOESM1_ESM.docx]
